# Valuing a Lifestyle Intervention for Middle Eastern Immigrants at Risk of Diabetes

**DOI:** 10.3390/ijerph15030413

**Published:** 2018-02-27

**Authors:** Sanjib Saha, Ulf-G. Gerdtham, Faiza Siddiqui, Louise Bennet

**Affiliations:** 1 Health Economics Unit, Department of Clinical Science (Malmö), Lund University, Medicon Village, Scheelevägen 2, SE-22381 Lund, Sweden; ulf.gerdtham@med.lu.se; 2 Center for Primary Health Care Research, Department of Clinical Sciences, Lund University/Region Skåne, Skåne University Hospital, SE-20502 Malmö, Sweden; faiza.siddiqui@med.lu.se (F.S.); louise.bennet@med.lu.se (L.B.); 3 Department of Economics, Lund University, SE-22363 Lund, Sweden; 4 Department of Clinical Sciences, Lund University, SE-20502 Malmö, Sweden

**Keywords:** willingness-to-pay (WTP), lifestyle intervention, immigrants, type 2 diabetes

## Abstract

Willingness-to-pay (WTP) techniques are increasingly being used in the healthcare sector for assessing the value of interventions. The objective of this study was to estimate WTP and its predictors in a randomized controlled trial of a lifestyle intervention exclusively targeting Middle Eastern immigrants living in Malmö, Sweden, who are at high risk of type 2 diabetes. We used the contingent valuation method to evaluate WTP. The questionnaire was designed following the payment-scale approach, and administered at the end of the trial, giving an ex-post perspective. We performed logistic regression and linear regression techniques to identify the factors associated with zero WTP value and positive WTP values. The intervention group had significantly higher average WTP than the control group (216 SEK vs. 127 SEK; *p* = 0.035; 1 U.S.$ = 8.52 SEK, 2015 price year) per month. The regression models demonstrated that being in the intervention group, acculturation, and self-employment were significant factors associated with positive WTP values. Male participants and lower-educated participants had a significantly higher likelihood of zero WTP. In this era of increased migration, our findings can help policy makers to take informed decisions to implement lifestyle interventions for immigrant populations.

## 1. Introduction

Lifestyle interventions with diet and physical exercise are effective in delaying and/or preventing the onset of type 2 Diabetes (T2D) in high-risk individuals, as shown in high-quality randomized trials all over the world [1]. Such interventions have also been shown to be cost-effective [2]. Middle Eastern immigrants living in Europe are at high risk of developing T2D [3], but there is a lack of evidence regarding the effectiveness of lifestyle interventions addressing this group. Given that such immigrants represent a growing proportion of the European and Swedish population, and that they exhibit a different cultural and socioeconomic background compared to the native population [3,4,5], there is an urgent need for preventive actions addressing this vulnerable population. For example, immigrants from the Middle East and their offspring living in Malmö, Sweden, have different diabetes-related risk factors such as a high prevalence of obesity and insulin resistance, sedentary lifestyles, and family history contributing to their excess risk of developing T2D compared to the native Swedish population [6]. In spite of their efficacy as a preventive strategy for T2D, lifestyle interventions designed for native populations may not achieve equivalent results in immigrant groups [7]. Immigrants not only differ in their lifestyle habits, but also face different cultural and social barriers when it comes to adopting a lifestyle modification. Gender-related barriers to physical activity, differences in perception of diabetes risk factors, traditional cooking practices, language barriers, and economic constraints are some of the factors motivating design of a culturally-adapted lifestyle intervention in this group [8,9]. Considering these differences, a culturally-adapted randomized controlled trial (RCT) of a lifestyle intervention (the Impact of Migration and Ethnicity on Diabetes in Malmö (MEDIM) study) was piloted among Middle Eastern immigrants at high risk of T2D living in Malmö, Sweden [10]. The intervention had significant beneficial effects on some risk factors of T2D such as increased insulin sensitivity, reduction on body weight, and reduction in low-density lipoprotein cholesterol in the intervention group compared to the control group [11].

There is only limited research on people’s willingness to participate in lifestyle intervention trials. Participation in trials within a clinical setting is usually free of charge, and participants often get an incentive to participate in the trial. However, if lifestyle interventions were to be implemented in the clinical practice, the participants would have to pay at least part of the program costs out-of-pocket. Taking into consideration this need for at least partial financing by the participants, it is worthwhile examining the amount of money that individuals would be willing to pay to participate in such a lifestyle intervention, as well as the factors that would affect their willingness to pay (WTP).

Few studies have estimated WTP to participate in a lifestyle intervention program for participants who have T2D or are at high risk of T2D. Van Gils et al. [12] and Veldwijk et al. [13] estimated WTP for participants who already had T2D, but not those at risk of T2D. Reed et al. [14] estimated WTP for patients at risk of T2D, but the intervention was hypothetical, and not actually experienced by the participants. Moreover, none of these studies estimated WTP among immigrant populations.

Once we have obtained a better insight into and knowledge of intervention-related factors that are crucial for participants’ WTP, recommendations can be made as to what type of program would most likely be preferred by its potential users. These recommendations can be taken into account when developing lifestyle-intervention trials, thus increasing their reach and hence the public health benefit.

The objective of this study was, therefore, to estimate the WTP of the participants’ for the lifestyle intervention and the factors that affect the WTP. 

## 2. Materials and Methods

### 2.1. The Intervention

The MEDIM intervention trial was conducted in the first half of 2015. Participants were recruited from the MEDIM baseline study [3], a population-based cross-sectional study recruiting Iraqi-born and Swedish residents of Malmo. The recruitment process and sample-size calculation has previously been described [10,11]. The inclusion criteria for the intervention trial was high body mass index (BMI) (≥28 kg/m^2^), large waist circumference (≥80 cm in women and ≥94 cm in men), or the presence of pre-diabetes, where pre-diabetes was defined as impaired fasting plasma glucose (6.1–6.9 mmol/L), impaired glucose tolerance (2-h glucose: 7.8–11 mmol/L), or impaired glucose regulation (impaired fasting as well as 2-h plasma glucose) [15]. The inclusion criteria corresponded to an increased risk of T2D [16]. The exclusion criteria were T2D, pregnancy, and mental or physical incapacity to participate in the study. 

The duration of the lifestyle intervention was four months. All participants were invited for three health examinations at the start, middle, and end of the trial. At the health examinations, anthropometric measurements were taken, blood samples were collected, and oral glucose tolerance tests were performed by trained study nurses. In addition, participants filled out questionnaires relating to their lifestyle habits, socio-economic status, mental health, and health-related quality of life. Randomization took place after the first health examination, allocating participants to the control or intervention group in a 1:1 ratio. Randomization was stratified by gender and facilitated by the random number generator in the SPSS statistical software package.

The lifestyle intervention comprised seven group sessions including one cooking class, and was adapted from the Diabetes Prevention Program (DPP) intervention [17]. The intervention was delivered by health coaches who had knowledge of Middle Eastern culture and were supported by a professional translator. The group sessions addressed understanding of the association between lifestyle, diabetes, and cardiovascular diseases as well as the barriers to lifestyle change, with the aim of empowering participants and increasing their self-efficacy for lifestyle change. The control group received treatment as usual i.e., they received brief written advice on increasing their physical-activity level and on following healthy dietary habits at the start, middle and end of the trial. This information was distributed along with the health examination results. Details of the intervention can be found elsewhere [11,18].

### 2.2. Willingness to Pay (WTP)

Willingness to pay (WTP) is a measure proposed in the framework of welfare theory for placing a monetary value on the welfare gain or loss of an intervention. The method was initially used to value public goods not subject to the market, but today it is extensively used in healthcare services [19]. Participants put a monetary value on participating in the intervention, considering that some form of welfare would be gained due to participation. If an individual states a high (low) WTP, then it can be inferred that the demand for the intervention is high (low) [20]. In the case of a RCT, if the intervention group’s WTP is higher than that of the control group, the intervention can be considered successful; that is, the participants were satisfied with the lifestyle intervention provided in the trial.

We used the contingent valuation (CV) method [20] to determine the maximum amount of money the participants would be willing to pay for the lifestyle intervention. After the end of the trial, participants were asked to complete a questionnaire to measure their WTP. The questionnaire was constructed following the payment card (PC) technique [21], which presents participants with a range of bid amounts in a vertical list from the lowest bid (top) to the highest bid (bottom) in increments. Participants were asked to indicate the amount that they were sure they would pay, the amount that they were sure they would not pay, and an amount representing their maximum WTP [22]. The chosen range ran from 50–1000 Swedish Krona (SEK) (1 U.S.$ = 8.52 SEK, 2015 price year) [23]. The questionnaire also captured whether or not participants were willing to pay any amount at all (positive WTP or zero WTP, respectively) to participate in this type of lifestyle intervention, and in the case of zero WTP response, the reason for not being willing to pay any amount. The WTP questionnaire is provided in the Supplementary File.

### 2.3. Variables Studied

Details regarding the collection of anthropometric and clinical measures and socioeconomic status have been described previously [11,18]. In the present article, we have included demographic variables such as age, sex, and marital status. Marital status was categorized as married (or living together) or single (or divorced/widowed). BMI was calculated by dividing body weight in kg by the square of body height in meters, and was categorized into <30 and ≥30. Education was categorized as high or low, with high education defined as having a college and/or university degree. 

Previous research suggests that acculturation, the process in which immigrants become gradually acculturated into the host culture by adopting its values, customs, and practices, can have an effect on healthcare utilization [24,25]. In this study, acculturation was captured by the duration of residence in Sweden (≤10 years or >10 years) and by the language used at home; the latter was categorized as speaking a mother tongue only or speaking both Swedish and mother tongue. Economic status was categorized as having had a job for the last 12 months, and the type of job. Types of job were dichotomized as self-employed or permanent/semi-permanent/non-permanent jobs. The variables are presented in Table 1.

### 2.4. Statistical Analyses

The statistical analyses were performed using version 14 of the Stata (StataCorp., College Station, TX, USA) software package. Differences between two groups were compared using an independent sample *t*-test for normally distributed or a Mann–Whitney U test for non-normally distributed continuous variables, and a chi-square test or Fisher’s exact test for categorical variables. The participants with zero WTP and positive WTP values, respectively, were identified. A descriptive analysis was used to present measures of the central tendency and dispersion with mean, median, and interquartile ranges for positive WTP. Logistic regression and linear regression techniques were used to capture socioeconomic and intervention-related factors that were crucial for zero WTP and positive WTP, respectively. Several models were developed including variables of interest. First, we wanted to see whether being in the intervention group affected the outcome i.e., univariate analysis (model 1). Thereafter, models were developed based on the physiological characteristics i.e., BMI (model 2), demographic characteristics (model 3), acculturation (model 4), economic status (model 5), and a model with all the variables of interest (model 6). In all the models being in the intervention group was included. Demand curves have been developed for both the intervention group and control group considering participants’ actual WTP value and the proportion of WTP for both groups following the guidelines of Emma Frew [22]. The Kolmogorov–Smirnov test has been performed to test the difference between the demand curve of the intervention group and the control group. Due to the non-normal distribution, the positive WTP values were transformed to natural logarithms in order to perform linear regression. The significance level was set at *p* ≤ 0.05 and standard error was provided.

### 2.5. Ethical Approval

The study protocol and informed consent form (in Arabic) were approved by the Ethical Review Board of Lund University, Sweden (Approval No. 2011/88).

## 3. Results

**Figure 1:**
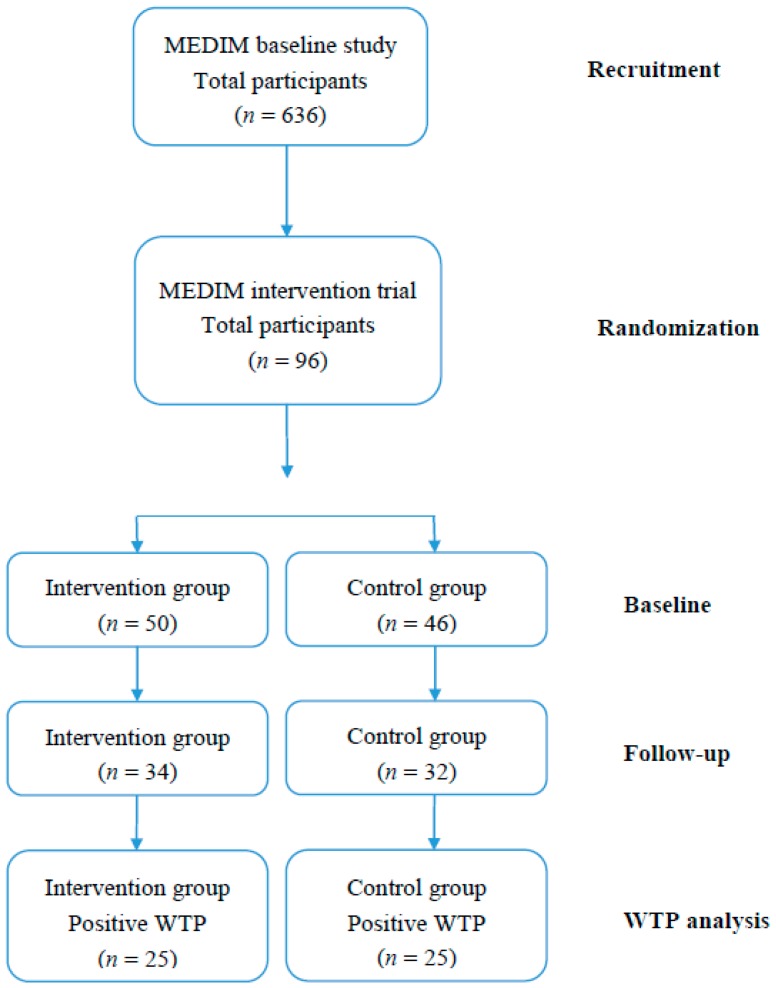
Flowchart of the MEDIM study participants.

A total of 96 participants were randomized to the intervention (*n* = 50) or control group (*n* = 46) after the first health examination. At the last visit 67 participants were present and 66 (34 in the intervention group and 32 in the control group) completed the WTP questionnaire. There were no significant differences between the intervention and control group regarding the variables studied (Table 1). A flow chart showing the participants throughout the trial and the questionnaire response rate is given in Figure 1. 

Zero WTP was reported by 24% of the participants, with no significant difference in prevalence between the control and intervention groups. Table 2 presents the results of logistic regression models predicting the likelihood of providing zero WTP values. Being in the intervention group had no effect on the likelihood of providing zero response (i.e., not wanting to pay), but male participants and low-educated participants had a significantly higher likelihood of providing zero response. The effect of education is no longer significant when controlling for all the variables.

Table 3 presents the summary data for the positive WTP values. The intervention group had a higher mean and median WTP than the control group. The value of the trimmed mean (which excludes 5% of observations equally at the two extremes of the distribution) indicates that the true mean was not influenced by small numbers of very high WTP values. We used the frequency distributions of the WTP values to create demand curves for both the intervention group and the control group; that is, curves which plot WTP on the horizontal axis against the cumulative proportion of the participants’ WTP on the vertical axis.

**Figure 2:**
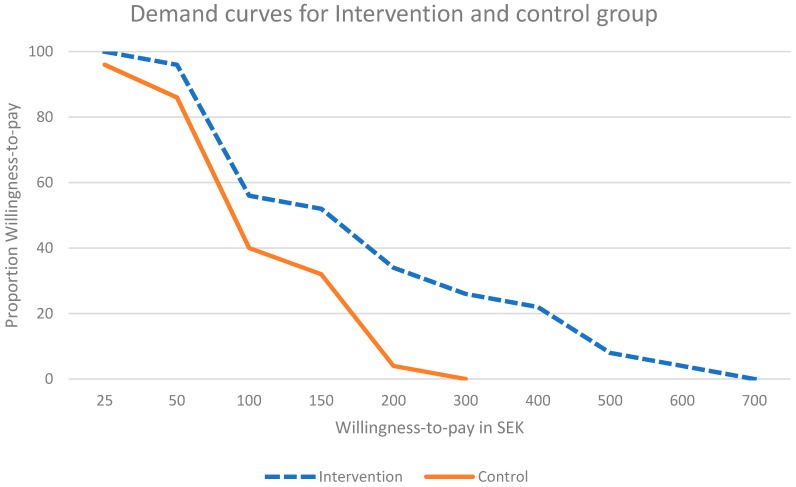
Willingness-to-pay demand curve.

The demand curves for the intervention group and the control group are given in Figure 2, showing clearly that the intervention group had a higher demand for the lifestyle intervention than the control group (Kolmogorov-Smirnov test, *p* < 0.05).

Table 4 presents the linear regressions explaining the positive WTP values. Being in the intervention group was a predictor of higher WTP. WTP was not affected by demographic characteristics such as age, sex, marital status, and educational status (model 3, Table 4), nor by BMI at baseline (model 2, Table 4). However, acculturation had an effect on WTP; participants using the Swedish language at home as well as their mother tongue were willing to pay more (model 4, Table 4). Participants who had had a job during the last 12 months were willing to pay more than those who were unemployed, and type of job also had a significant effect, with self-employed participants being willing to pay more. Being in the intervention group and type of job remained significant after controlling all the variables of interest (model 6).

## 4. Discussion

This study estimated the effectiveness, in terms of participants’ WTP to participate, of a lifestyle intervention exclusively designed for and piloted among Middle Eastern immigrants at high risk of T2D. Our findings reveal that the intervention group participants were willing to pay more to participate in the lifestyle intervention than the control group.

The median WTP value among the intervention group participants was 200 SEK per month. The figure is higher than that found in a Dutch study of lifestyle intervention for T2D patients [13], but lower than the figure reported in a study in the USA of participants at high risk of T2D [14]. Different methods were used to estimate WTP in these studies, but one possible explanation for the difference could be that the participants in the US study were highly educated and had yearly incomes higher than the national average [14].

We also found that women were more willing to provide a positive WTP value than men (Table 2). This is consistent with the popular belief that a woman would pay more attention to and worry more about her weight than a man [26]. Education had a positive effect on positive WTP, which is in line with a study on WTP for physical activity among the general Swedish population [27]. The reason for this may be that more highly educated participants are more health literate; that is, they have increased knowledge of risk factors and the harmful aspects of T2D, and more positive attitudes towards a healthy lifestyle in the Swedish context [28]. However, the effect of education was no longer significant while controlling all the variables of interest. It is not clear whether the reason is for controlling all the variables or low power due to small sample size and many variables in a single-regression model (model 6).

We also found that acculturation was a predictive factor of higher WTP, which is in line with previous studies suggesting that acculturation has an effect on utilization of healthcare [24,25]. For instance, studies on social capital have shown that individuals with a high level of acculturation have higher trust in authorities [29]. A higher trust in authorities could have contributed to a higher WTP in the present study, as the intervention was conducted by researchers from a reputable university in Sweden. Addressing acculturation could help policy makers, not only in Sweden but also in other countries, in designing lifestyle interventions and recruiting participants from other cultural backgrounds.

Researchers have previously suggested that income predicts higher WTP for lifestyle interventions [12,13,14]. We had no information on the income of our participants, but instead used the presence of a job in the last 12 months and type of job as a proxy for income. Although having a job was not a significant predictor of WTP, the type of job (self-employed) had a significant effect on WTP besides being in the intervention group.

It is interesting to note that being in the intervention group or control group did not have an effect on the likelihood of not being willing to pay anything (zero WTP). However, being in the intervention group had a significant effect on positive WTP even after controlling all the variables. This could be interpreted to mean that the intervention group participants were happy with the lifestyle intervention that they had received, and thus were willing to pay more than the control group. In other words, it could be perceived as reflecting the effectiveness of this culturally-adapted lifestyle intervention.

The use of the CV method seems appropriate for this study [30]. This is because CV surveys are conducted when the commodity being valued such as lifestyle intervention is not available in the market. However, there is some concern that the CV method may overestimate WTP as it relies on hypothetical rather than actual behavior, as suggested in a meta-analysis of 59 studies [31]. In this study, we used the CV method to capture the real experience of the intervention group instead of a hypothetical scenario, in order to minimize the bias.

The PC is a commonly-accepted tool for capturing WTP, due both to its suitability and to its advantages and convenience compared with other formats and other methods of estimating WTP for healthcare interventions [22]. However, this technique is affected by range bias; that is, the respondent may be influenced by the range of values chosen for the PC question design [32]. In order to minimize any possible bias that might have been induced by the range of the PC, the WTP questionnaire was designed in two phases, the first providing a broad range and the second providing a blank space instead of a range for capturing the maximum WTP (Supplementary File).

The WTP questionnaire was administered at the end of the trial, following an ex post (after) perspective [30]. It could be argued that an ex ante (before) perspective would be more appropriate for WTP analysis as respondents did not receive the intervention at baseline measurement time and hence might provide a true response to WTP. However, we wanted the experience of the participants to be real, instead of presenting a hypothetical scenario. Therefore, it seemed appropriate to use the ex post perspective and ask the participants at the end of the intervention, in order to minimize the uncertainty regarding the health outcome. With an ex post perspective, the change of welfare has happened; and the WTP, in being elicited from the utility level after the change has happened, is thus an equivalent variation [33]. Moreover, since MEDIM is a RCT, it is reasonable to consider that the intervention and control groups would likely have had the same WTP at baseline. However, the study might have been affected by bias stemming from “warm glow” feelings [34]. The intervention group participants might have felt some moral obligation (a “warm glow”) towards the intervention providers, and due to this might have given a higher WTP value which did not reflect their true preference [22]. Research is limited on how to minimize the “warm glow” bias for RCTs in an ex post perspective [35].

Researchers have suggested determining the characteristics of respondents by providing zero WTP values; in this study, these respondents constituted 24% of the participants [22]. We therefore performed analyses with both logistic and linear regression techniques. Although it would have been feasible to estimate a single Tobit model or a two-part model for the full sample instead of two models for zero values and positive values, respectively, earlier research has found that this two-part specification performs better with WTP data [36,37]. One might argue that a stepwise regression would have been an alternative instead of 6 different models. We were interested in factors that can capture physiological, demographic, acculturation and economic status and, thus, we chose to present 6 different models. However, a stepwise regression showed that being in the intervention group and type of job were still statistically significant factors for positive WTP (results are presented in Tables S1 and S2).

It is hard to distinguish between a true zero response and a protest response in a CV study. Protesters are the respondents who may state zero WTP, even though they care about the intervention, because they feel that it is someone else’s responsibility to pay. Therefore, we eliminated the zero response, considering that zero was not a credible answer, as suggested by Diamond and Hausman [38].

The limitations of the study must be considered, and the results should be interpreted with caution. First, the sample size was smaller than we had expected while planning the study design [10] both for clinical and health economic outcomes. Despite our best effort to recruit as many participants as possible—sending a mail invitation followed by repeated telephone contacts by an Arabic-speaking study nurse, providing all information in both Swedish and Arabic, and offering flexible appointments, an easily accessible study center, and incentives—the participation rate was not optimal. Therefore, the generalization of these findings in other settings may be limited. One possible solution for future lifestyle interventions would be to use the referral of high-risk individuals by general physicians (GPs) instead of an open invitation, as in an earlier Norwegian study [39]. Moreover, in comparison with non-participants, the participants had lower BMI and a larger proportion of physically active individuals, indicating that those who were comparably healthier or fitter might have been more likely to participate in the trial and thus the study might have “healthy volunteer bias”. Second, there was a high dropout rate, as many participants were not present at the last check-up. A dropout analysis showed that those who dropped out were younger and reported lower levels of physical activity at baseline compared to those who remained in the study. It thus seems that only those who were highly motivated finished the study, and so selection bias might have occurred. However, there were no significant differences between the intervention and control group in respect of dropout rate, sex, or BMI (results not shown here).

The regression models have a low explanatory capacity, as shown in the adjusted R-squared value, which can be described as limited. This would not be improved by adding new explanatory variables to the models, because objective variables cannot fully explain personal choice or the perception of wellbeing, as recognized by other authors [40,41]. Factors underlying willingness to participate in a lifestyle intervention should be studied more extensively, especially among the immigrants.

The intervention group participants were given financial support (500 SEK) after buying a gym membership, clothes or shoes for physical exercise. One might ask whether providing incentives might have triggered them to stipulate higher WTP values than the control group. We argue that the benefits of the intervention, not the incentives, motivated them to provide higher WTP. The intervention group achieved healthy outcomes such as lower body weight and improved low-density lipoprotein, insulin sensitivity [11], reduced dietary intake of energy, carbohydrate, sucrose and fat [18] and overall health-related quality of life. However, it may not be possible to identify what triggered the intervention group to provide higher WTP; it could have been the short-term benefit of the intervention (weight loss), the long-term benefit of the intervention (delay of T2D onset), or the financial incentive.

The strengths of this study were as follows. First, this is the only study in which WTP was estimated for a RCT of a lifestyle intervention exclusively designed for Middle Eastern migrants at high risk of T2D. Second, the detailed description of the context, the intervention data collection, analyses and findings can contribute to the methodological robustness of a WTP study.

It would be interesting for future research to explore the unobservable factors related to WTP for immigrants, using a large sample size and a longer duration intervention. A qualitative analysis such as in-depth interviews, focus-group discussion or consensus technique might be helpful for exploring factors that affect not only the WTP but also the effectiveness, participation and adherence of lifestyle intervention of the immigrant population. Moreover, an economic evaluation could be performed considering the cost of providing the intervention, in order to estimate whether it is economically worthwhile to implement this type of lifestyle intervention for Middle Eastern immigrants at high risk of developing T2D.

## 5. Conclusions

In this era of increased migration from the Middle East to the European Union, our findings can help policy makers to take informed decisions about implementing lifestyle interventions to prevent or delay the costly disease of T2D, and thus improve the overall health status of immigrant populations and save valuable societal resources.

## Figures and Tables

**Table 1:** Baseline characteristics of the Impact of Migration and Ethnicity on Diabetes in Malmö (MEDIM) participants answering the willingness-to-pay (WTP) questionnaire.

Variables	Control Group (*n* = 32)	Intervention Group (*n* = 34)	*p* Value
Age ^a^	48.78 (8.54)	50.76 (10.40)	0.402
Body mass index (BMI) ^b^			
≥30	17 (53%)	18 (53%)	0.98
<30	15 (47%)	16 47%)	
Sex ^b^			
Male	17 (53%)	19 (56%)	0.46
Female	15 (47%)	15 (44%)	
Education ^b^			
High	22 (69%)	29 (85%)	0.11
Low	10 (31%)	5 (15%)	
Marital status ^b^			
Married or living together	26 (81%)	29 (85%)	0.66
Single	6 (19%)	5 (15%)	
Job in last 12 months ^b^			
Yes	18 (56%)	16 (47%)	0.46
No	14 (44%)	18 (53%)	
Job type ^c^			
Self-employed	3 (9%)	4 (12%)	0.53
Permanent/temporary	29 (91%)	30 (88%)	
Migration duration ^b^			
≤10 years	7 (22%)	12 (35%)	0.23
>10 years	25 (78%)	22 (65%)	
Language(s) spoken at home ^b^			
Mother tongue only	23 (72%)	28 (82%)	0.31
Swedish and mother tongue	9 (28%)	6 (18%)	

^a^ mean (standard deviation) and *t*-test; ^b^ frequency (percentage) and chi-square test; ^c^ frequency (percentage) and Fisher’s exact test.

**Table 2:** Logistic regression predicting zero willingness to pay (zero value = 0).

Variable	Model 1		Model 2		Model 3		Model 4		Model 5		Model 6	
	OR (SE)	*p*	OR (SE)	*p*	OR (SE)	*p*	OR (SE)	*p*	OR (SE)	*p*	OR (SE)	*p*
Intervention group	0.777 (0.45)	0.66	0.777 (0.45)	0.66	0.49 (0.33)	0.30	0.85 (0.51)	0.79	0.85 (0.50)	0.79	0.49 (0.36)	0.33
BMI at baseline (<30)			0.85 (0.49)	0.78							1.07 (0.7)	0.91
Age					0.96 (0.03)	0.23					0.95 (0.04)	0.17
Sex (male)					4.1 (0.17)	0.04					0.22 (1.7)	0.047
Education (high)					4.6 (3.43)	0.04					4.21 (3.5)	0.08
Marital status (single)					0.50 (0.42)	0.42					0.43 (0.4)	0.36
Language at home (mother tongue)							2.40 (1.9)	0.29			2.85 (2.7)	0.27
Migration duration (>10 years)							1.12 (0.72)	0.86			1.71 (1.4)	0.51
Job in last 12 months (no)									0.39 (0.26)	0.16	0.54 (0.44)	0.45
Types of job (self-employed)									2.26 (2.25)	0.416	1.72 (1.93)	0.63
Adjusted R squared	0.02		0.00		0.20		0.06		0.03		0.27	

Reference groups are in parentheses; BMI = body mass index; OR = odds ratio; *p* = *p*-value; SE = standard error.

**Table 3:** Summary statistics for positive willingness-to-pay values (SEK).

Statistics	All Participants (*n* = 50)	Intervention (*n* = 25)	Control (*n* = 25)
Mean ^a^	171.50	216	127
Standard error of mean	19.09	33.63	13.84
Mean (5% trimmed)	154	199	124
Median	100	200	100
Mode	100	100	100
Coefficients of skewness	0.529	0.689	0.390
Percentiles			
25	100	100	75
50	100	200	100
75	200	250	200
90	300	540	200

^a^ *p*-value = 0.035, Mann-Whitney U-test between intervention and control group.

**Table 4:** Linear regression for positive willingness-to-pay values (natural logarithms) *.

Variable	Model 1		Model 2		Model 3		Model 4		Model 5		Model 6	
	b (SE)	*p*	b (SE)	*p*	b (SE)	*p*	b (SE)	*p*	b (SE)	*p*	b (SE)	*p*
Intervention group	0.463 (0.18)	0.014	0.429 (0.18)	0.021	0.455 (0.19)	0.023	0.548 (0.18)	0.004	0.43 (0.17)	0.013	0.38 (0.18)	0.045
BMI at baseline (<30)			−0.28 (0.18)	0.120							−0.21 (0.18)	0.25
Age					0.003 (0.01)	0.76					0.004 (0.01)	0.63
Sex (male)					0.024 (0.19)	0.90					−0.28 (0.18)	0.14
Education (high)					0.017 (0.25)	0.90					−0.21 (0.25)	0.40
Marital status (single)					0.20 (0.26)	0.45					0.32 (0.24)	0.18
Language at home (mother tongue)							0.412 (0.2)	0.045			0.22 (0.22)	0.31
Migration duration (>10 years)							0.204 (0.2)	0.320			−0.06 (0.24)	0.79
Job in last 12 months (no)									−0.05 (0.17)	0.771	−0.09 (0.2)	0.65
Types of job (self-employed)									−0.926 (0.29)	0.003	−1.06 (0.32)	0.002
Adjusted R squared	0.10		0.14		0.04		0.16		0.26		0.27	

Reference groups are in parentheses; BMI = body mass index; b = coefficient; *p* = *p* value; SE = Standard error.

## Supplementary Materials

The following are available online at http://www.mdpi.com/1660-4601/15/3/413/s1, Willingness-to-pay questionnaire, Table S1: Stepwise logistic regression predicting zero willingness to pay (zero value = 0), Table S2: Stepwise linear regression for positive willingness-to-pay values (natural logarithms).
