# Supplementary material for: Valuing a Lifestyle Intervention for Middle Eastern Immigrants at Risk of Diabetes
Source: Int J Environ Res Public Health. 2018 Feb 27;15(3):413. doi: 10.3390/ijerph15030413 (PMC5876958; doi:10.3390/ijerph15030413)
Supplement: Supplementary file 1 [file ijerph-15-00413-s001.pdf]

Supplementary Materials: Valuing a Lifestyle Intervention for Middle Eastern Immigrants at Risk of Diabetes

Sanjib Saha, Ulf-G Gerdtham, Faiza Siddiqui and Louise Bennet

Willingness-to-pay questionnaire

1. Are you willing to pay (WTP) some money to attend this type of program?
- Yes (go to question 3)
- No
2. What is your reason for saying "No"?
- I do not like this type of program
- I cannot afford to pay
- Other (please specify)
3. Please tick (✔) the amount that you are sure you would pay per month (SEK) to participate in the program.
- 100
- 200
- 300
- 400
- 500
- 600
- 700
- 800
- 900
- 1000
4. Please tick (✔) the amount that you are sure you would not pay per month (SEK) to participate in the program.
- 100
- 200
- 300
- 400
- 500
- 600
- 700
- 800
- 900
- 1000
5. What is your maximum willingness to pay to participate in the program?
   Answer: __________________ SEK/month

Table S1. Stepwise logistic regression predicting zero willingness to pay (zero value = 0).

Variable	*OR*	*p*
Sex (male)	0.305	0.060
Education (high)	3.02	0.090
Adjusted R squared	0.086	

Reference groups are in parentheses; *OR* = odds ratio; *p* = *p*-value.

Table S2. Stepwise linear regression for positive willingness-to-pay values (natural logarithms).

**Variable**	***b***	***p***
Intervention group	0.425	0.012
Types of job (self-employed)	−0.952	0.001
Adjusted R squared	0.27	

Reference groups are in parentheses; *b* = coefficient; *p* = *p* value.

© 2018 by the authors; licensee MDPI, Basel, Switzerland. This article is an open access article distributed under the terms and conditions of the Creative Commons by Attribution (CC-BY) license (http://creativecommons.org/licenses/by/4.0/).
